# Supplementary material for: On demand laser-induced frequency tuning of coherent magnons in a nanometer-thick magnet at room temperature
Source: Nat Commun. 2026 Jan 6;17:145. doi: 10.1038/s41467-025-66707-7 (PMC12775049; doi:10.1038/s41467-025-66707-7)
Supplement: Supplementary file 1 — Supplementary Information [file 41467_2025_66707_MOESM1_ESM.pdf]

Supplementary Information: On demand laser-induced frequency tuning of coherent magnons in a nanometer-thick magnet at room temperature

Volker Wiechert[1], Hanchen Wang[2], William Legrand[2], Pietro Gambardella[2], David Breitbach[3], Philipp Pirro[3], Michaela Lammel[1], Andrea Meo[4], Giovanni Finocchio[5] and Davide Bossini[1]

[1]Department of Physics and Center for Applied Photonics, University of Konstanz, D-78457 Konstanz, Germany

[2]Department of Materials, ETH Zurich, Zurich 8093, Switzerland.

[3]Fachbereich Physik and Landesforschungszentrum OPTIMAS, Rheinland-Pfälzische Technische Universität Kaiserslautern-Landau, D-67663 Kaiserslautern, Germany

[4]Department of Electric and Information engineering, Politecnico di Bari, I-70125 Bari, Italy

[5]Department of Mathematical and Computer Sciences, Physical Sciences and Earth Sciences, University of Messina, I-98166 Messina, Italy

SQUID characterization

To determine the laser-induced demagnetization, the temperature dependence of the magnetization must be characterized. For this purpose, SQUID magnetometry is carried out at different temperatures with an MPMS-3 from Quantum Design. The raw data were corrected for parasitic signals of the substrate and sample holder to extract the signal stemming from the BiYIG thin film. From the out-of-plane hysteresis loops, the saturation magnetization at each temperature can be extracted.

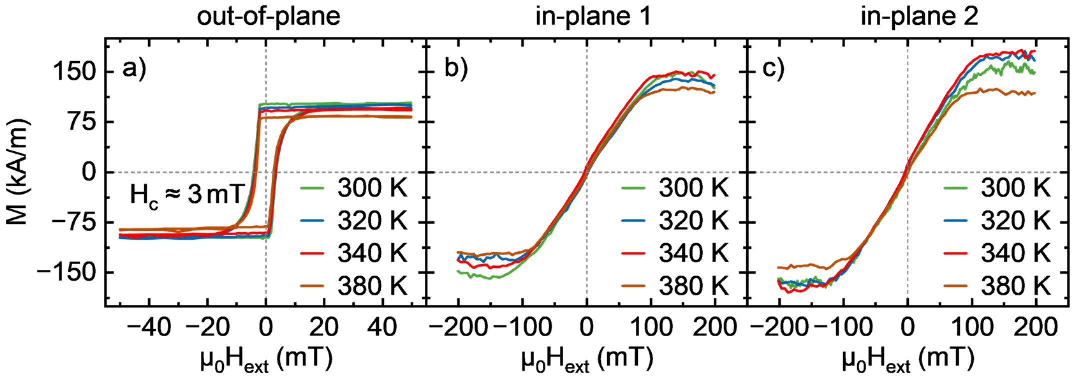

Supplementary Fig. 1: SQUID magnetometry measurements. Magnetization VS external field SQUID magnetometry measurements for a) out-of-plane magnetic field and b), c) two different in-plane field directions. All data were acquired at different sample temperatures. We note that "in-plane 1" and "in-plane 2" indicate two orthogonal directions in the plane of the sample parallel to the sample edges.

The absence of hysteresis in the in-plane SQUID loop shown in Supplementary Fig. 1 indicates that the magnetization is not stabilized in-plane, i.e., the system does not possess an in-plane easy axis. This observation is consistent with the presence of a strong perpendicular magnetic anisotropy, where the easy axis is oriented out-of-plane.

Polarization-independent effect of the pump polarization

Supplementary Fig. 2 reports a series of measurements as a function of the pump polarization. The photo-induced spin-dynamics does not display any dependence on the pump polarization of the pump beam.

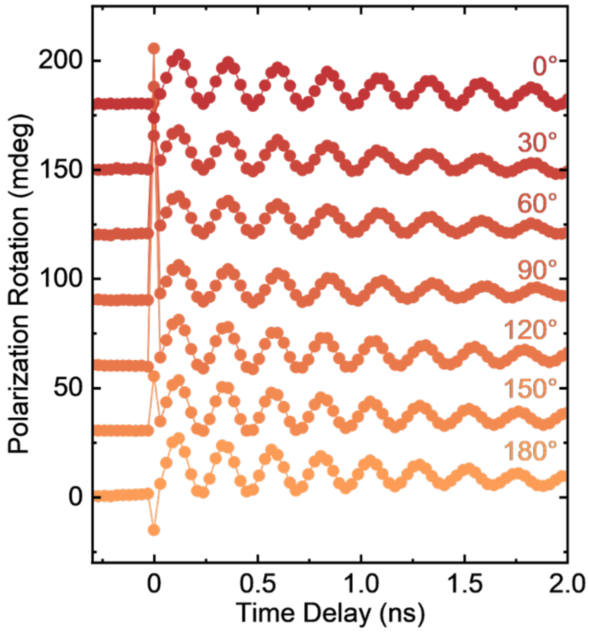

Supplementary Fig. 2: Pump polarization dependence of the rotation of the polarization. The probe beam was linearly polarized along the horizontal plane. The measurements were performed setting the excitation fluence to 3 mJ/cm^2 and applying an external magnetic field of 200 mT.

Observation of coherent signal in multidomain state

An in-plane field biases the local oscillation axis of the magnetization to a specific direction. Consequently, coherent signal from different domains with the same magnetization direction dominates on the signal generated by domains, in which the magnetization is differently oriented. To tackle this point also experimentally, we have performed measurements as a function of the magnetic field, especially addressing the multi-domain regime. The evaluation of the amplitude (fit-parameter *a* in S(1)) of the magnon oscillations, measured as a function of the in-plane field (Supplementary Fig. 3) confirms our statement: the field biases the local axis of the magnetization to a specific direction. Accordingly, as the in-plane field is reduced, the signal amplitude decreases and approaches zero for 0 mT (Supplementary Fig. 3b)). This is also shown by atomistic simulations performed for decreasing magnetic fields down to 1mT. In agreement with the experimental results, simulations show how the signal amplitude decreases approaching zero as the in-plane field is reduced (Supplementary Fig. 3b)).

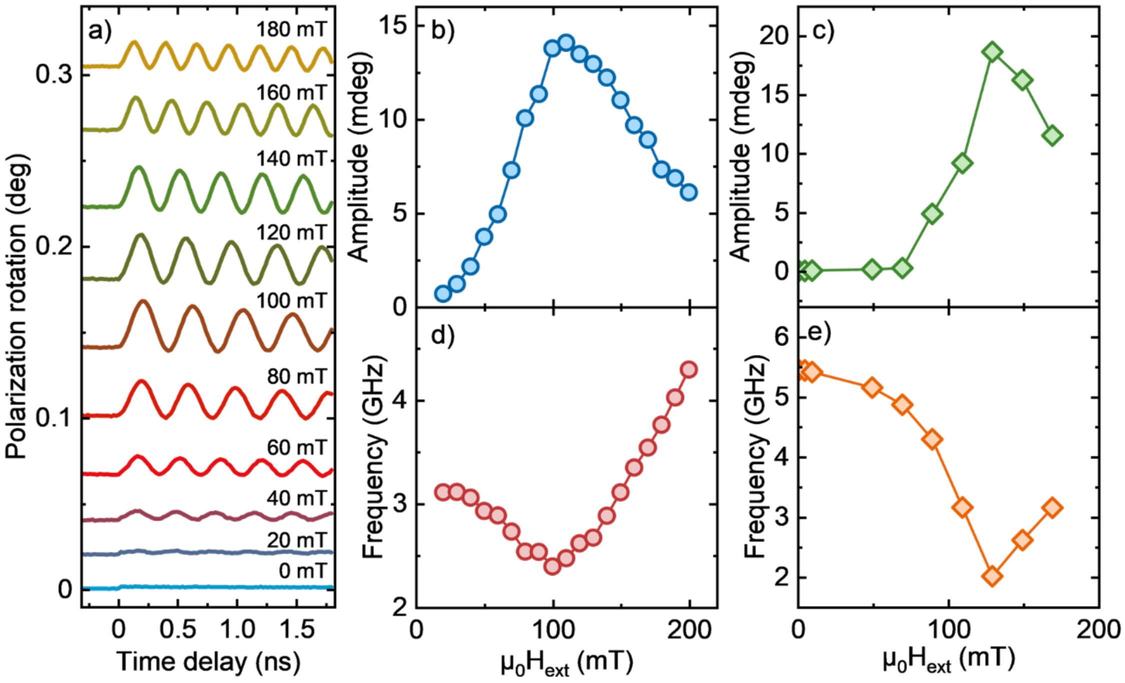

Supplementary Fig. 3: External field dependence of oscillation amplitude and frequency. a) Selection of time-resolved transient probe polarization rotations for different external fields ranging from 180 to 0 mT. The data are horizontally offset for better visibility. Oscillations are absent only at 0 mT. b) Extracted amplitude of the magnetization oscillation for different external magnetic fields and c) from atomistic simulations. As the field approaches 0 mT the amplitude vanishes. d) Corresponding oscillation frequencies to the presented time-resolved measurements and e) the frequencies obtained from simulations. Pump and probe beams were horizontally polarized. The excitation fluence in these measurements was set to 0.5 mJ/cm^2 in experiments and 2 mJ/cm^2 in simulations.

Our results are also in agreement with the literature that demonstrates that two main modes should be measurable in the multidomain state[1–3], originating either from the domain volume or from the dynamics of the walls. The frequency of the domain volume mode decreases as the applied magnetic field increases. This trend is consistent with our observation, suggesting that our scheme is sensitive only to the domain volume mode. We believe that the dynamics of the wall cannot be monitored, because the volume of the walls is much more limited than the volume of the domains. Our experimental method is sensitive to an average response throughout the sample volume, so the response of the domain dominates.

Full hysteresis loop measurements

Supplementary Fig. 4 presents the oscillation frequency (panels (a) and (c)) and amplitude (panels (b) and (d)) of the photoinduced coherent magnons measured along the hysteresis loop, as requested by the Reviewer. The loop was recorded from –200 to 200 mT (blue) and back (red) at fluences of 0.5 and 8 mJ/cm^2. The direction of the field sweep and the sign of th field do not produce a systematic detectable. difference A comparison with the simulation data likewise reveals no significant deviation.

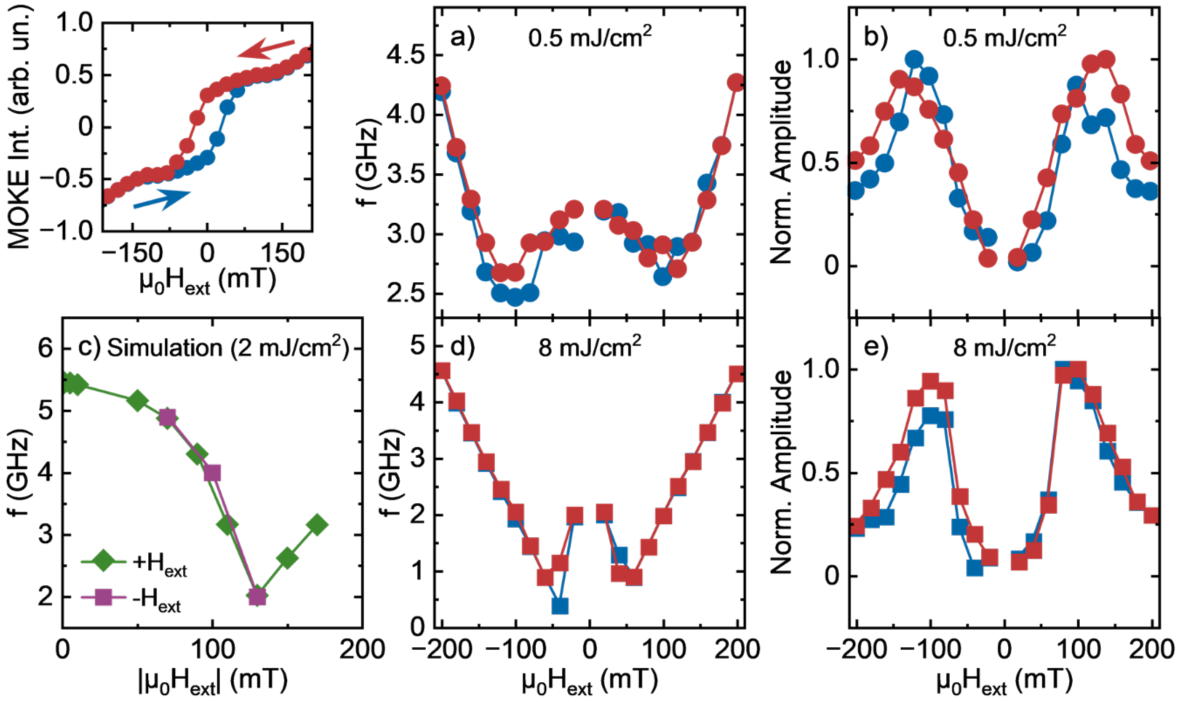

Supplementary Fig. 4: Frequency and oscillation amplitude for full hysteresis loop. The leftmost panel indicates the sweep direction. The corresponding data in (a)–(d) are color-coded accordingly: data from –200 to 200 mT are shown in blue, and data from the return path are shown in red. a), c) Oscillation frequencies of the photoinduced coherent magnons and b), d) corresponding normalized amplitudes of the oscillation for the full hysteresis loop for the fluences 0.5 and 8 mJ/cm^2. Pump and probe beams were horizontally polarized.

Data analysis

Fitting procedure

The pump-probe time traces are fitted using the equation

$$\Delta\theta_z(t) = a\,e^{-t/\tau}\cos(2\pi f t + \phi) + a_{BG}e^{-t/\tau_{BG}} + c_{BG}, \qquad \text{S(1)}$$

where the first term on the righthand side describes the exponentially damped part of the magnetization precession with a frequency f and initial phase ϕ. The amplitude a corresponds to the precession angle. The second and third term of the righthand side describe the incoherent background signal and an offset in the data, respectively.

The frequencies extracted from the time domain fit are used in the following to determine the anisotropy. For this purpose, the frequencies are adjusted to a fixed fluence depending on the applied external field using a Kittel-fit from Eq. M(2). The reduction of magnetization discussed in the previous section are used for this calculation.

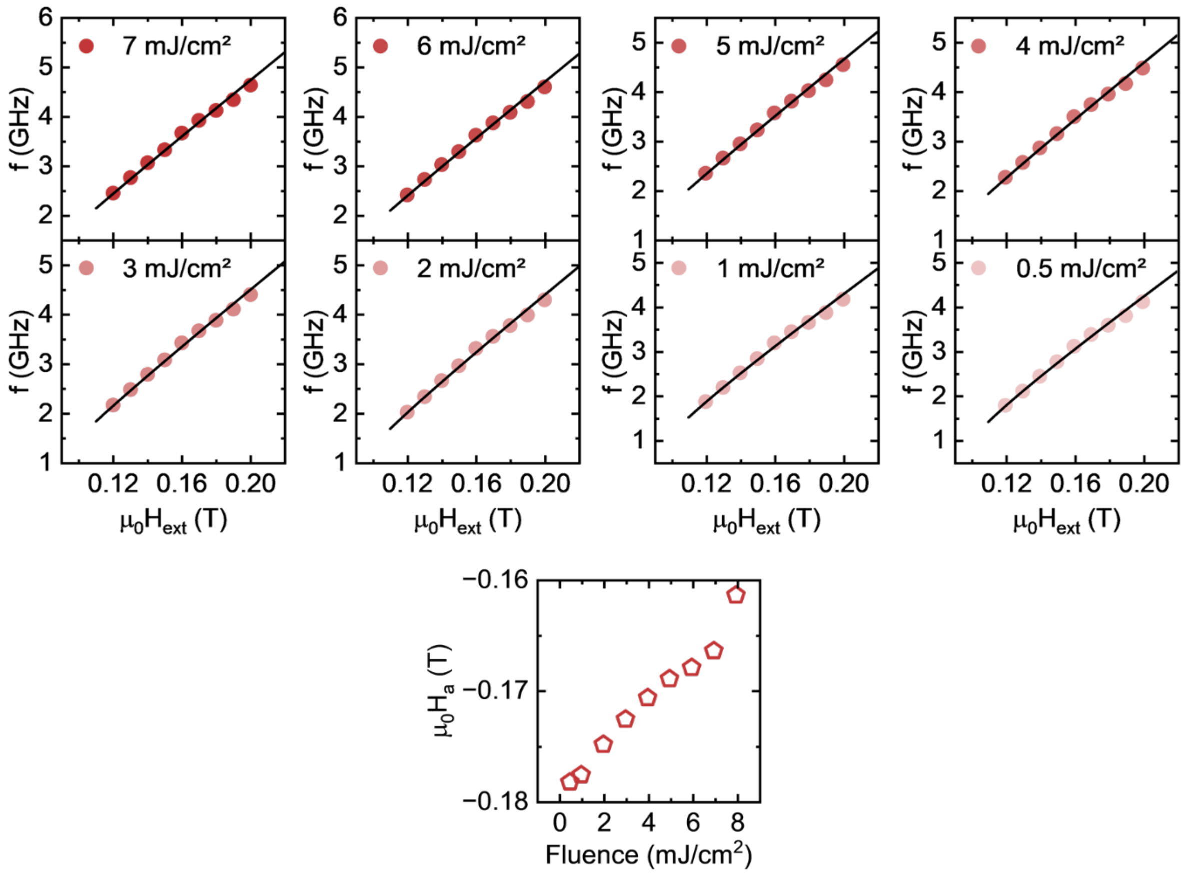

Supplementary Fig. 5: Determination of the photoinduced anisotropy changes. Frequencies from the time domain (data fitted with Eq. S(1)) and the corresponding fits with Eq. M(2) to determine the anisotropy for different fluences for external fields in saturation. The values of the anisotropy determined from the fit to the data with Eq. M(2) is shown in the lower panel.

Magnon Damping

The magnon damping mainly originates from spin–lattice interactions. To assess the role of laser heating, we extract the magnon dephasing time $\tau(F)$ from the data fits S(1) as a function of pump fluence. The corresponding effective damping

$$\alpha_{eff}(F) = \frac{1}{2\pi \cdot \tau(F) \cdot f(F)}, \qquad \text{S(2)}$$

is shown in Supplementary Fig. 6 panel b). It increases linearly up to $5\ \mathrm{mJ/cm^2}$ before saturating. This behavior is attributed to laser-induced incoherent phonons that scatter with spin waves, reducing their lifetime until a bottleneck occurs near this fluence. Since the damping already incorporates the frequency shift, the data reflect a fluence-dependent reduction of the resonance quality factor. A microscopic explanation is beyond the scope of the present manuscript. However, calculations show that ellipticity effects on damping are negligible under the initial anisotropy field of the sample. This effect becomes even smaller, if the anisotropy field is reduced, which is exactly what happens once the sample is illuminated by the pump beam.

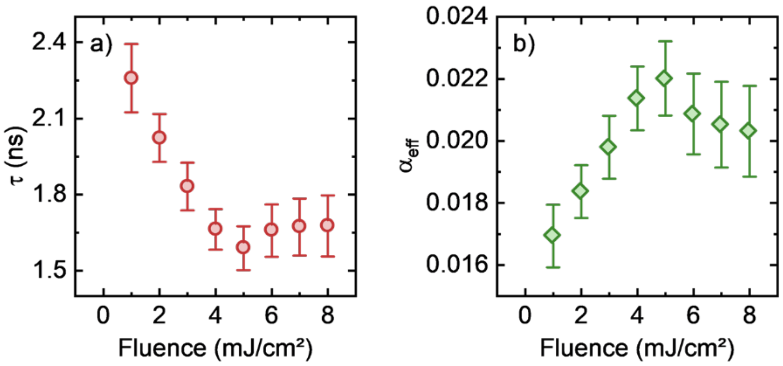

Supplementary Fig. 6: Lifetime and effective damping. Lifetime time (left) and effective damping (right) as a function of the pump fluence. Measurements were performed under an external magnetic field of 200 mT. Domain structure in the equilibrium state

Domain structure in the equilibrium state

To gain a deeper understanding of the static behavior of the sample's magnetization, Kerr microscopy was performed in the polar MOKE geometry under various in-plane magnetic fields. For this purpose, the Magneto-Optical Kerr Microscope from *Evico Magnetics* was used. The structure was illuminated with white light, and image acquisition was carried out using the integrated microscope equipped with a polarizer–analyzer setup. To enhance the Kerr contrast, the sample was initially saturated using an in-plane magnetic field of −180 mT. The reference image recorded during this process was subsequently subtracted from all other captured images.

Identification of the magneto-optical detection effect

Supplementary Fig. 7 reports a series of measurements as a function of the probe polarization. No change in the rotation of the polarization is observed, which allows us to identify the origin of the magneto-optical signal with the Faraday effect. Our experimental set-up is thus sensitive to the out-of-plane component of the magnetization precession [4].

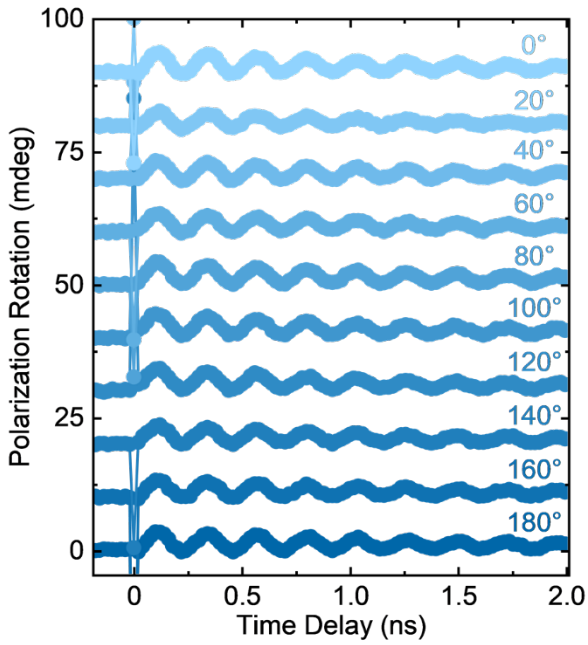

Supplementary Fig. 7: Probe polarization dependence of the rotation of the polarization. The pump beam was linearly polarized along the horizontal plane. The measurements were performed setting the excitation fluence to 1 mJ/cm^2 and applying an external magnetic field of 200 mT.

Determination of the opening angle from the pump-probe data

An objective quantification of the laser-induced perturbation of the magnetic ground state demands to express the detected Faraday rotation in terms of the precession angle (see Supplementary Fig. 8).

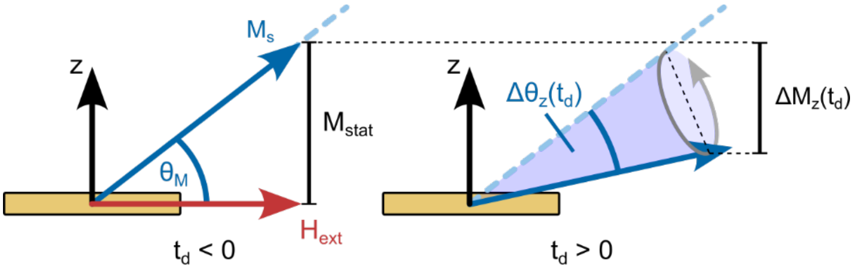

Supplementary Fig. 8: Determination of the precession angle of the magnetization a, Equilibrium orientation of the magnetization. The static magnetization M_{stat} along the z-axis is determined prior to the laser excitation. After the excitation the time-resolved measurement is only sensitive to the change in the z-component of the magnetization oscillation $\Delta M_z(t_d)$. The combination of static (**a**) and dynamics (**b**) measurement enables the determination of the oscillation opening angle $\Delta\theta(t_d)$.

In the static case, the saturation magnetization M_S forms an angle θ_M relative to the film plane, which can be calculated analytically [5] . The physically measurable quantity in the experiment corresponds to the projection of the saturation magnetization onto the z-axis, M_{stat}. In the pump-probe experiment, the projection of the magnetization deviating from the static position, $\Delta M_z(t_d)$, is measured. Applying trigonometry, the precession angle of the magnetization $\Delta\theta(t_d)$ can be determined in the following way:

$$M_{\text{stat}} - \Delta M_z(t_d) = M_S \sin\left(\theta_M - \Delta\theta_z(t_d)\right) \quad \text{S(3)}$$

$$\Rightarrow \Delta\theta_z(t_d) = \arcsin\left[\frac{M_{\text{stat}}}{M_S}\right] - \arcsin\left[\frac{M_{\text{stat}}}{M_S} - \frac{\Delta M_z(t_d)}{M_S}\right],$$

where $\sin(\theta_M) = \frac{M_{\text{stat}}}{M_S}$ was used in the last passage.

Laser-induced demagnetization

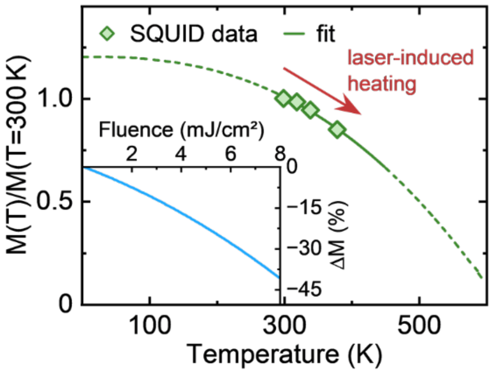

Supplementary Fig. 9: Estimation of laser-induced demagnetization. Temperature dependent magnetization curve with modified Bloch function as a guide to the eye of the magnetization change due to laser-induced heating. Data extracted from the SQUID hysteresis loops. Inset: Calculated laser-induced demagnetization from the temperature dependence of the magnetization for different excitation fluences[45].

[45]Based on the measured hysteresis loops (see Supplementary Fig. 1), the temperature-dependent change of the magnetization $M(T)$ in Bi:YIG in the relevant range (solid line) can be described using the first leading term of the conventional Bloch model [6]:

$$\frac{M(T)}{M(T = 300K)} = M_0 \cdot \left(1 - aT^{5/2}\right), \qquad \text{S(4)}$$

with the parameters $M_0 = 1.205$ and $a = 1.039 \times 10^7\ K^{-5/2}$. The laser-induced temperature change ΔT of the sample can be estimated using the following equation:

$$\Delta T(F) \approx F \cdot \frac{\alpha}{\rho \cdot d \cdot c_W} \approx F \cdot \frac{21.8\ \text{K}}{\text{mJ/cm}^2}, \qquad \text{S(5)}$$

Where F is the used laser fluence, $\alpha = 0.1390$ is the absorbance, $\rho \approx 6.4\ \text{g/cm}^3$ the density of the Bi:YIG-layer with thickness $d = 20\ \text{nm}$ and heat capacity $c_W \approx 0.5\ \text{J/gK}$

[7]. By combining both equations S(4) and S(5), the demagnetization induced by laser heating can be calculated. It can be expressed as:

$$\Delta M(F) = M_0\left(1 - a(T + \Delta T)^{5/2}\right) - M_0\left(1 - aT^{5/2}\right). \qquad \text{S(6)}$$

Atomistic spin dynamics simulations

Simulations were performed using the open-source VAMPIRE atomistic spin dynamics code [8]. The BiYIG lattice is described by classical unit-length spins $\mathbf{S}_i$ on the 20-site garnet unit cell comprising the a-Fe^{3+}-ions with tetrahedral coordination and the d-Fe^{3+} ions with octahedral coordination, in a crystal of lattice constant $a_0 = 1.2376\ \text{nm}$ [9].The system Hamiltonian is defined as:

$$\mathcal{H} = -\frac{1}{2}\sum_{ij} J_{ij}\, \mathbf{S}_i \cdot \mathbf{S}_j - \sum_i k_u (\mathbf{S}_i \cdot \boldsymbol{e}_{\text{ani}})^2 - \sum_i \mu_{s,i}\, \mathbf{B} \cdot \mathbf{S}_i \qquad \text{S(7)}$$

where J_{ij} are isotropic exchange constants between spins i and j at sites i and j, respectively, k_u is the uniaxial anisotropy constant along the easy-axis direction $\boldsymbol{e}_{\text{ani}}$, $\mu_{s,i}$ is the spin magnetic moment of spins on site i, $\mathbf{B}$ the external magnetic field.

We describe the exchange interactions in BiYIG via nearest-neighbor exchange constants $J_{ad} = -9.60 \times 10^{-21}\text{J}\,(\sim -6.0\text{ meV})$, $J_{dd} = -3.24 \times 10^{-21}\text{J}\,(\sim -2.0\text{ meV})$ and $J_{aa} = -0.92 \times 10^{-21}\text{J}\,(\sim -0.6\text{ meV})$ [10,11] where the subscripts aa, dd, and ad stand for the a-Fe^{3+}- a-Fe^{3+} , d-Fe^{3+}- d-Fe^{3+} and a-Fe^{3+}- d-Fe^{3} interactions, respectively. Despite the frustration due to all coupling being antiferromagnetic, the dominant intersublattice J_{ad} makes the antiparallel state the ground state. We consider a magnetic moment $\mu_{s,i} = 5\,\mu_B$ for both Fe sublattices, in agreement with other works in the literature [11] , and a damping $\lambda = 2 \times 10^{-4}$. We also include a small uniaxial anisotropy $k_u = 2.32 \times 10^{-24}\text{J}$ to account for the PMA in BiYIG.

The spin dynamics is described by the atomistic Landau-Lifshitz-Gilbert equation based on the Hamiltonian of Eqn. S(7):

$$\frac{d\mathbf{S}_i}{dt} = -\frac{\gamma}{1+\lambda^2}\mathbf{S}_i \times \boldsymbol{H}_{\text{eff,i}} - \frac{\gamma\lambda}{1+\lambda^2}\mathbf{S}_i \times \mathbf{S}_i \times \mathbf{B}_{\text{eff}} \qquad \text{S(8)}$$

where $\gamma = 1.76 \times 10^{11}\text{ rad s}^{-1}\text{T}^{-1}$ is the gyromagnetic ratio and λ is the atomistic Gilbert damping constant. The local effective field $\mathbf{B}_{\text{eff}} = (-1/\mu_s)\,\partial\mathcal{H}/\partial S_i + \boldsymbol{\xi}_i$ includes, in addition to the internal energy terms of the Hamiltonian given in Eqn. S(7), the stochastic term $\boldsymbol{\xi}_i$. ξ_i describe the interaction of the spin system with a classical heat-bath characterized by $\langle\xi_i(t)\rangle = 0$ and $\langle\xi_i(t)\xi_j(t')\rangle = \delta_{ij}\delta(t-t')2\lambda\mu_s k_B T/\gamma$, where k_B is the Boltzman constant.

Ultrafast temperature dynamics is described via a two-temperature model coupling electronic and phononic baths. The coupled rate equations are:

$$C_e\frac{dT_e}{dt} = -G_{ep}\,(T_e - T_p) + P(t) \qquad \text{S(9)}$$

$$C_p\frac{dT_p}{dt} = G_{ep}\,(T_e - T_p)$$

Here, C_e and $C_p = 3 \times 10^6\text{ J m}^{-3}\text{K}^{-1}$ are the electronic and phononic heat capacities, respectively[12] , G_{ep} the electron-phonon coupling and $P(t)$ describes the laser heating pulse with a Gaussian profile. In an insulating system, we can expect $C_p \approx 0\text{ J m}^{-3}\text{K}^{-1}$ since there are no free electrons. However, we can still utilize this approach if we consider magnon-like properties. If we do so, we can estimate G_{ep} by considering the magnon specific heat C_m ($\approx 1 \times 10^4\text{ J m}^{-3}\text{K}^{-1}$ of YIG-systems[13] via the expression $G_{ep} = C_m C_p/(\tau_{mp}(C_m + C_p))$, where τ_{mp} is the magnon-phonon relaxation time, which we can estimate in the range of few picoseconds, giving $G_{ep} \approx 1 \times 10^{16}\text{ W m}^{-3}\text{K}^{-1}$.

Throughout this work we consider a $24.752\text{ nm} \times 24.752\text{ nm} \times 24.752\text{ nm}$ system with periodic boundary conditions to the in-plane directions, i.e., x- and y-axes, to simulate a thin film, as in the experiments. The spin dynamics simulations are performed integrating a Heun-type solver with a 0.1 fs timestep. Simulations are performed at 300 K and, for

the laser-induced dynamic simulations, the system is equilibrated for 10 ns under the external field before the heat pulse is applied.

1. Tacchi, S. *et al.* Rotatable magnetic anisotropy in a Fe0.8Ga0.2 thin film with stripe domains: Dynamics versus statics. *Phys. Rev. B* **89**, 024411 (2014).

2. Camara, I. S. *et al.* Magnetization dynamics of weak stripe domains in Fe–N thin films: a multi-technique complementary approach. *J. Phys.: Condens. Matter* **29**, 465803 (2017).

3. Liu, C. *et al.* Current-controlled propagation of spin waves in antiparallel, coupled domains. *Nat. Nanotechnol.* **14**, 691–697 (2019).

4. Zvezdin, A. K. & Kotov, V. A. *Modern Magnetooptics and Magnetooptical Materials*. (1997). doi:10.1201/9780367802608.

5. Krivosik, P. & Patton, C. E. Hamiltonian formulation of nonlinear spin-wave dynamics: Theory and applications. *Phys. Rev. B* **82**, 184428 (2010).

6. Maier-Flaig, H. *et al.* Temperature-dependent magnetic damping of yttrium iron garnet spheres. *Phys. Rev. B* **95**, 214423 (2017).

7. Kazei, Z. A., Kolmakova, N. P. & Sokolov, V. I. *Garnets*. (1991). doi:10.1007/10041375_59.

8. Evans, R. F. L. *et al.* Atomistic spin model simulations of magnetic nanomaterials. *J. Phys.: Condens. Matter* **26**, 103202 (2014).

9. Xie, L.-S. *et al.* First-principles study of exchange interactions of yttrium iron garnet. *Phys. Rev. B* **95**, 014423 (2017).

10. Cherepanov, V., Kolokolov, I. & L'vov, V. The saga of YIG: Spectra, thermodynamics, interaction and relaxation of magnons in a complex magnet. *Phys. Rep.* **229**, 81–144 (1993).

11. Barker, J. & Bauer, G. E. W. Thermal Spin Dynamics of Yttrium Iron Garnet. *Phys. Rev. Lett.* **117**, 217201 (2016).

12. Zeuschner, S. P. *et al.* Standing spin wave excitation in Bi : YIG films via temperature-induced anisotropy changes and magneto-elastic coupling. *Phys Rev B* **106**, 134401 (2022).

13. Rezende, S. M., Rodríguez-Suárez, R. L., Ortiz, J. C. L. & Azevedo, A. Thermal properties of magnons and the spin Seebeck effect in yttrium iron garnet/normal metal hybrid structures. *Phys. Rev. B* **89**, 134406 (2014).
